# The effect of ondansetron intravenous administration to caloric intake for patients of gynecological surgery

**DOI:** 10.1186/s40780-026-00557-3

**Published:** 2026-02-20

**Authors:** Eiji Horita, Mitsuo Kaneshima, Yasuhisa Kobayashi, Masaki Sano, Yoshie Takebayashi, Kenichiro Saito, Yoshinori Munemoto, Kazuko Mitsuya, Hiroyuki Satomi, Kumiko Hosokawa, Tetsuji Kurokawa, Koichi Shimo, Satoshi Shine

**Affiliations:** 1 Department of Pharmacy, Fukui-ken Saiseikai Hospital, 7-1, Funabashi, Wadanaka-cho, Fukui, 918-8503 Japan; 2 Department of Obstetrics and Gynecology, Fukui-ken Saiseikai Hospital, 7-1, Funabashi, Wadanaka-cho, Fukui, 918-8503 Japan; 3 Department of Nutrition, Fukui-ken Saiseikai Hospital, 7-1, Funabashi, Wadanaka-cho, Fukui, 918-8503 Japan; 4 Department of Surgery, Fukui-ken Saiseikai Hospital, 7-1, Funabashi, Wadanaka-cho, Fukui, 918-8503 Japan; 5 Department of Anesthesiology, Fukui-ken Saiseikai Hospital, 7-1, Funabashi, Wadanaka-cho, Fukui, 918-8503 Japan; 6 Nutrition Support Team, Fukui-ken Saiseikai Hospital, 7-1, Funabashi, Wadanaka-cho, Fukui, 918-8503 Japan

**Keywords:** Postoperative nausea and vomiting, Ondansetron, Caloric intake

## Abstract

**Background:**

Postoperative nausea and vomiting can be a serious issue in reducing caloric intake for patients in the early stage after surgery. In Japan, ondansetron injection is now approved by insurance as a countermeasure against postoperative nausea and vomiting, but the number of reports on its effects regarding caloric intake is limited in the early stage following surgery, and opinions about the effects are divided. Thus, we examined how the effects of ondansetron administration during surgery influence caloric intake starting the day after surgery.

**Methods:**

We examined 65 patients who received a 4mg injection of ondansetron during gynecological surgery under epidural anesthesia, in comparison to 51 patients who did not receive any antiemetic. Our study was to compare the amount of caloric intake the day after surgery.

**Results:**

The patient group who received an ondansetron injection showed higher caloric intake (1364.1 ± 55.9 vs 1188.3 ± 63.1 kcal; ANCOVA, *p* = 0.045). A significant increase in caloric intake was observed in patients with an Apfel Score of 3 (807 ± 62 vs 593 ± 76 kcal; *p* = 0.031).

**Conclusions:**

Our study indicated that ondansetron administration during gynecological surgery may have a positive effect on increasing postoperative caloric intake one day after surgery.

## Background

Postoperative nausea and vomiting (PONV) is one of the causes of significantly reduced caloric intake in the early stage after surgery [1]. While PONV is a common adverse event during the postoperative recovery stage, it could greatly affect patient’s quality of life (QOL) and nutritional management. PONV is not limited to the day after surgery but occurs at certain frequencies even a few days after surgery [2–4]. In our facility’s recovery ward, we often observe patients with insufficient caloric intake due to PONV.

Ondansetron is effective for prevention of PONV as a 5-HT3 receptor antagonist [5–7]. Japan started insurance coverage of ondansetron beginning August 2021. Of course, patients strongly wish to avoid pain and PONV [8]. For anesthesiologists also preventing the occurrence of PONV is a crucial issue [9]. We consider it great progress that we can administer ondansetron during surgery in terms of perioperative management.

Reports on how ondansetron affects postoperative caloric intake at the early stage after surgery are, however, quite limited and opinions are inconclusive [1, 10, 11]. In this study, we examined how the effects of ondansetron administration during surgery influences caloric intake starting the day after surgery.

## Patients and methods

### Research design and subjects

**Fig. 1:**
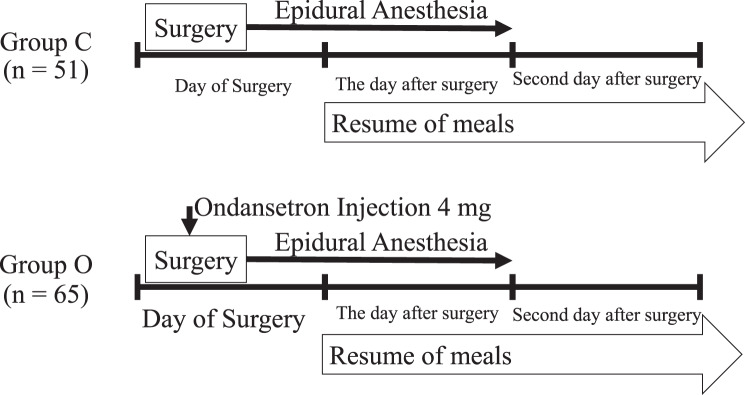
Protocol of scheduled ondansetron 4 mg injection. Only Group O patients received ondansetron injection 4 mg during surgery in order to avoid postoperative PONV. Both groups (Group O and Group C) continuously received epidural anesthesia one day after surgery. Patients in both groups resumed meals the day after surgery

This study is a retrospective study of patients who had gynecological surgery under epidural anesthesia from April 2021 to March 2022. We grouped 65 patients who received a 4 mg ondansetron injection during surgery (Group O) and 51 patients who did not receive any antiemetic (Group C). Based on the clinical pathway, caloric intake with meals started one day after surgery (postoperative day 1)（Fig. 1）.

### Data collection

We collected data retrospectively from electronic medical records. We examined patients’ background information, including age, body weight, surgical method (vaginal surgery, laparoscopic surgery, or open surgery), Apfel Score, doses of fentanyl and local anesthetic adjusted for body weight, and operation time. Apfel Score was assessed using the four established risk factors: female sex, non-smoking status, history of PONV and/or motion sickness, and postoperative opioid use [12–14]. The four risk factors of the Apfel Score were routinely documented in the medical records based on a standardized preoperative oral interview, and the Apfel Score (0–4) was recorded as the sum of these factors; these data were extracted retrospectively for this study. “Non-smoker” was operationally defined at our institution as smoking cessation for ≥1 year; this operational definition was used to ensure consistent classification, considering the potential long-term effects of nicotine on neural receptor changes [14]. A history of PONV was defined as at least one prior episode after anesthesia. Motion sickness was recorded only when currently present; childhood-only motion sickness was excluded. Patients who did not receive postoperative opioids were excluded per the prespecified eligibility criteria; therefore, postoperative opioid use was applicable to all included patients.

### Evaluation of PONV

We evaluated PONV existence based on doctors’ orders in the electronic medical records. To be more specific, we determined that a patient experienced PONV if one of the instructions was given by doctors: (1) prescription of antiemetic, and/or (2) instruction for reduction or cancelation of epidural anesthesia due to suspicion of PONV. Because this was a retrospective study, mild symptoms that did not lead to a prescription of an antiemetic or an instruction for reduction or cancelation of epidural anesthesia may not have been captured.

### Evaluation of dietary intake

We evaluated oral caloric intake one day after surgery. Caloric intake was calculated based on nursing records. Patients’ meal preparation generally complied with the clinical pathway, but occasionally they were modified based on the patient’s taste or medical conditions (such as diabetic). When meal types were changed, the calorie content corresponding to the modified meal was used. Meal intake was routinely recorded by nurses separately for staple foods and side dishes using a 0–10 “tenths” score (0 = none, 10 = all). Each one-point increase corresponded to a 10% increment of the served portion. Estimated intake was calculated as (prescribed calories × recorded consumption score/10), and the total caloric intake was obtained by summing the calculated intake for staple foods and side dishes. In addition, we calculated oral intake percentage (%) as (total caloric intake/prescribed meal calories) × 100 on one day after surgery. For a threshold-based analysis, low oral intake was defined as <25% (severe poor intake) and <50% (insufficient intake) of prescribed meal calories.

## Statistical analysis

We used G*Power to calculate sample size, assuming detection power 0.80, significance level as 0.05, effect size (Cohen’s d) as 0.46 (medium or average). As a result, total 154 samples, each group of 77 samples, were needed. As for statistical analysis, we used Statistical Package for the Social Sciences (SPSS) version 24.0 (SPSS Inc. Chicago, IL). We used Fisher’s exact test, Wilcoxon’s rank sum test, and covariance analysis (ANCOVA). In ANCOVA, we set Apfel Score, amount of ropivacaine (Anapeine®) and fentanyl and operation time as covariates which could potentially influence calorie intake. We set significance level *p < 0.05* in order to determine statistical significance.

## Results

### Patients’ background

There was no statistical difference between Group O and Group C in terms of age, weight, surgery methods, Apfel Score, amount of fentanyl, and operation time. However, there was significantly less amount of ropivacaine in Group O (*p = 0.002*) (Table 1).

**Table 1:** Patients’ background

	Group C	Group O	p
Number of cases	51 (44.0%)	65 (56.0%)	
Patients’ age: mean, years	51.0 ± 1.7	52.4 ± 1.6	0.678^a)^
Body weight: mean, kg	59.4 ± 2.0	59.2 ± 1.5	0.911^a)^
Surgical Method			0.874^b)^
Vaginal surgery	13	14	
Laparoscopically assisted surgery	2	2	
Open surgery	36	49	
Apfel Score (2 factors)	3	5	1.000^b)^
Apfel Score (3 factors)	30	39	1.000^b)^
Apfel Score (4 factors)	18	21	0.843^b)^
Amount of Fentanyl/weight (μg/kg)	7.92 ± 2.10	7.56 ± 2.88	0.372^a)^
Amount of Ropivacaine/weight (mg/kg)	4.60 ± 1.07	4.05 ± 0.81	0.002^a)^
Operation Time (min)	94.0 ± 6.7	91.5 ± 4.5	0.876^a)^

Data were expressed as mean ± standard error and number of patients as appropriate. a)Used Wilcoxon rank sum test for statistical analysis. b)Used the Fisher exact test

### Effect on PONV

**Fig. 2:**
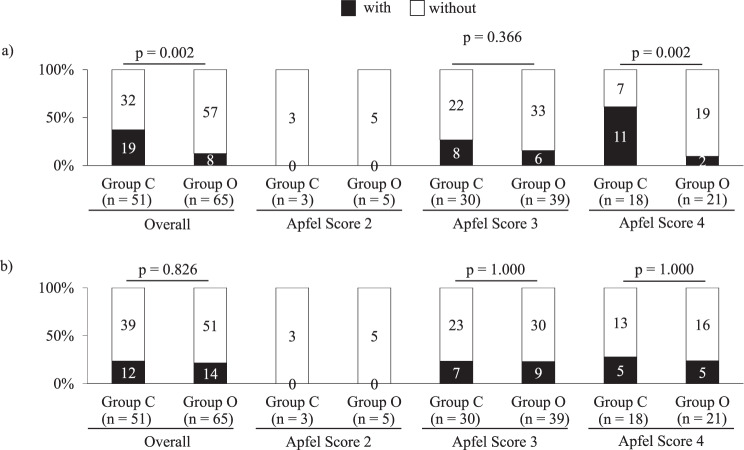
Conditions of postoperative PONV. This comparative figure shows whether or not (“with” or “without”) there were countermeasures against PONV on the day of and the day after surgery: **a**) indicates the day of surgery and **b**) indicates one day after surgery. Countermeasures against PONV include usage of antiemetic and/or reduction or cancellation of epidural anesthesia

On the day of surgery, PONV was significantly lower in Group O than in Group C in the overall analysis, and the suppression effect was also significant among patients with all four Apfel factors (Fig. 2a). In contrast, no significant between-group difference was observed one day after surgery (Fig. 2b).

### Effect on oral caloric intake

**Fig. 3:**
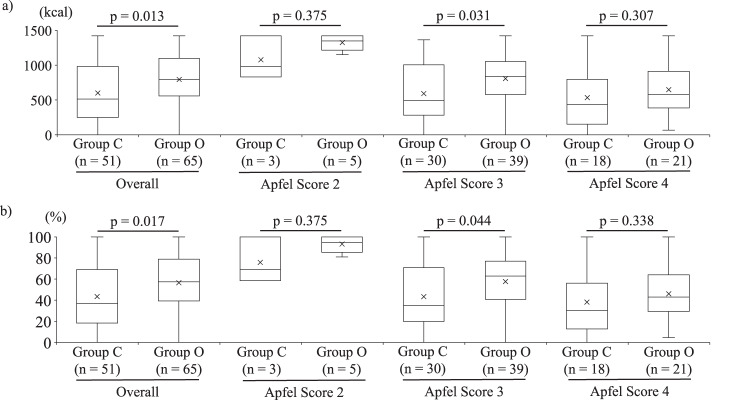
Postoperative oral intake on the day after surgery. (**a**) total calorie intake (kcal) in Group C and Group O. (**b**) oral intake percentage (%), calculated as the percentage of prescribed meal calories consumed. Box-and-whisker plots show overall data and stratification by Apfel score 2–4. Used Wilcoxon rank sum test for statistical analysis

Total caloric intake was higher in Group O than in Group C one day after surgery (Fig. 3a). Oral intake percentage (%) was also higher in Group O, particularly among patients with an Apfel score of 3 (Fig. 3b).

### Supplementary evaluation by Covariance Analysis

Since the sample size may be insufficient in this study, we did covariance analysis (ANCOVA) in order to adjust influential elements that could affect caloric intake. As a result, Group O, compared to Group C, showed higher caloric intake one day after surgery (F = 4.131, *p = 0.045*). The average caloric intake in Group O was 1364.1 ± 55.9 kcal while it was 1188.3 ± 63.1 kcal in Group C. The difference between the two groups was 175.8 kcal (95% confidence interval [4.3, 347.3])（Table 2）. Also, when we evaluated the influence of covariates, Apfel Score gave negative influence significantly to caloric intake (F = 7.386, *p = 0.008*, regression coefficient B = −196.8, 95% CI [−340.4, −53.3]). On the other hand, the amount of ropivacaine and fentanyl and operation time did not show any significant influence（Table 3）. A threshold-based analysis of low oral intake (<25% and <50%) is shown in Table 4.

**Table 2:** Result of covariance analysis (Difference of caloric intake with or without ondansetron injection)

Factors	F Value	P Value	Standard Error of Average ± Caloric Intake (kcal)	Mean Difference (Ondansetron - Control), kcal	95% Confidence Interval
Group C	-	-	1188.3 ± 63.1	175.8	[4.3, 347.3]
Group O	4.131	0.045	1364.1 ± 55.9	-	-

**Table 3:** Effects of covariates on covariance analysis

Elements	F Value	P Value	Regression Coefficient B	95% Confidence Interval
Apfel score	7.386	0.008	−196.8	[−340.4, −53.3]
Amount of Ropivacaine	0.248	0.619	−28.4	[−141.4, 84.6]
Amount of Fentanyl	0.652	0.421	16810.9	[−24469.4, 58,091.2]
Operation Time	1.170	0.282	−1.06	[−3.02, 0.89]

**Table 4:** Threshold-based analysis of oral intake achievement on postoperative day 1

Intake threshold	Group C (*n* = 51)	Group O (*n* = 65)	p
<25% (severe poor intake)	18 (35.3%)	11 (16.9%)	0.031b)
<50% (insufficient intake)	31 (60.8%)	28 (43.1%)	0.064b)

Low oral intake was defined as <25% (severe) and <50% (insufficient) of prescribed calories. b)Used the Fisher exact test

## Discussion

The purpose of this study was to determine the effect of ondansetron administered during gynecological surgery on postoperative caloric intake. Conventionally, the preventive effect of ondansetron on PONV is widely recognized. Our quantitative study focused on the point that such known preventive effect could expand to postoperative caloric intake.

In Group O, patients who received ondansetron during gynecological surgery showed significantly higher numbers of oral caloric intake one day after surgery. In addition to absolute caloric intake, we evaluated oral intake percentage (%) relative to prescribed meal calories and found a consistent tendency toward improved intake in the ondansetron group. This suggests that ondansetron may be clinically beneficial in reducing low oral intake early after surgery, although not all intake-related endpoints may show statistical significance. It was especially true to the high-risk patients that applied three factors from Apfel Score. This result indicates that suppression of PONV facilitates meal intake after surgery, and promotes improvement of nutritional condition. The standard diet provided at our hospital on the first postoperative day contains approximately 1400 kcal. The average difference of 175.8 kcal corresponds to approximately 13%, suggesting a potentially clinically relevant improvement in early postoperative oral intake. It is particularly true that increased protein intake plays a vital role in maintaining immune function and helping with wound healing [15]. Nutritional intake at the earlier stage after surgery could also contribute to reducing the risk of infection and shortening hospital stays [16, 17]. Administration of ondansetron could enable the patient to expedite resuming regular meals and drinking water even on the same day of surgery [10]. This too can be a positive element in promoting earlier nutritional intervention. This study could support those clinical efficacies. On one hand, there was a report on ondansetron which did not influence meal intake after surgery. The report was a comparison of breakfast and lunch intake one day after surgery [11]. Although our results were in line with this finding and ondansetron did not demonstrate a clinically meaningful effect on meal volume when evaluated using our threshold-based criteria, our quantitative analysis clarified that it contributed to increasing total caloric intake. This may represent a favorable aspect for promoting earlier nutritional intervention. Taken together, these observations underline the need for more structured and insurance-supported perioperative nutritional management.

Furthermore, as a result of covariance analysis, we recognized that the higher the Apfel Scores, the less the caloric intake becomes. It indicates that a patient with higher PONV risk tends to have more difficulty in caloric intake. We confirmed that ondansetron administration alleviates this tendency. The finding indicates the necessity of individualized anti-PONV strategies for a patient with higher risk. It also indicates that ondansetron administration is effective for postoperative nutritional management. On the other hand, the amount of ropivacaine or fentanyl, and operation time did not show significant influence on caloric intake, and we think that the relation between PONV and caloric intake could be strongly influenced by the use of antiemetic and patient’s individual risk profile.

This study is one of the rare research studies that quantitatively evaluated the influence of antiemetic administration during surgery on postoperative caloric intake. We believe that our study can provide a valuable new perspective in postoperative management.

## Research limitations

We recognize the following six limitations in this study. 1) This study did not achieve the planned sample size (154 subjects: 77 per group) estimated beforehand using G*Power. The actual sample size was 116 subjects (65 in the O group and 51 in the C group), which may have resulted in reduced statistical power and an increased risk of Type II error. Therefore, our findings, particularly non-significant results and subgroup analyses, should be interpreted with caution. 2) The sample patients were all females, but we did not look into the elements that could affect PONV such as menstrual cycle and menopause [18, 19]. 3) Our study is a retrospective study, and evaluation of PONV depends solely on electronic medical records. PONV assessment was based on doctors’ orders for antiemetic prescription or epidural reduction/discontinuation, which could lead to missed mild PONV cases and information bias due to physician variability. In addition, PONV was evaluated only on the day of surgery and on the following day; therefore, symptoms persisting for 48–72 hours postoperatively may not have been fully captured, although epidural anesthesia is typically discontinued by the morning of postoperative day 2 at our institution. Because patient-reported outcomes (NRS/scores) were not available in this retrospective study, future studies should combine patient-reported outcomes (NRS/scores) with quantitative food intake records to better evaluate the relationship between symptoms and postoperative intake. 4) Since postoperative pain intensity was not recorded, it could not be included as a covariate, leaving residual confounding possible. Although surgical techniques were comparable between groups (Table 1), unmeasured perioperative factors such as postoperative pain may have influenced early oral intake. While postoperative pain is routinely managed at our institution with nonsteroidal anti-inflammatory drugs such as loxoprofen or acetaminophen during gynecological surgery, this study could not quantitatively assess the effect of postoperative pain on oral intake. In addition, in other clinical settings at our institution, the combination of non-opioid analgesics has been reported to effectively alleviate postoperative pain [20]. 5) Our operational definition of “non-smoker” (≥1-year smoking cessation) reflects institutional practice and may differ from definitions used in other studies, which could affect reproducibility. 6) Caloric intake was calculated using a standardized method based on prescribed dietary calories and recorded intake ratios (0–10 scale) for staple foods and side dishes. However, because intake ratios were derived from routine nursing records rather than research-grade methods (e.g., weighed food records), some measurement error may remain. Such measurement error could have attenuated observed between-group differences. In addition, because detailed nutrient composition and oral fluid intake were not consistently available in the retrospective records, we could not evaluate protein intake, oral fluid intake, or overall nutritional balance.

Due to the limited sample size, this study is exploratory in nature. Despite these limitations, we believe our findings provide preliminary clinical value by showing quantitatively the influence of antiemetics during surgery on postoperative nutritional intake.

## Conclusions

This study suggests that administering ondansetron during gynecological surgery may be associated with improved caloric intake on the first postoperative day. The effect was observed in high-risk patient groups, indicating potential benefits as a new intervention strategy. We would like to continue more studies incorporating patients’ subjective evaluation index for more practical and highly reproducible evidence.

## Data Availability

The datasets generated and analyzed during the current study are not publicly available due to privacy concerns and institutional policy but are available from the corresponding author on reasonable request.

## Abbreviations

PONV: Postoperative nausea and vomiting
QOL: Quality of life
SPSS: Statistical Package for the Social Sciences
